# Isolation of retinal progenitor and stem cells from the porcine eye

**Published:** 2007-06-29

**Authors:** Ping Gu, Laura Jayne Harwood, Xiaohong Zhang, Mildred Wylie, William James Curry, Tiziana Cogliati

**Affiliations:** 1 Centre for Vision Sciences, Queens University Belfast, United Kingdom; 2 Veterinary Sciences Division, Department of Agriculture and Rural Development Northern Ireland, Belfast, United Kingdom

## Abstract

**Purpose:**

Retinal progenitor cells (RPCs) and retinal stem cells (RSCs) from rodents and humans have been isolated and characterized in vitro. Transplantation experiments have confirmed their potential as tools for cell replacement in retinal degenerative diseases. The pig represents an ideal pre-clinical animal model to study the impact of transplantation because of the similarity of its eye to the human eye. However, little is known about porcine RPCs and RSCs. We aimed to identify and characterize in vitro RPCs and RSCs from porcine ocular tissues.

**Methods:**

Cells from different subregions of embryonic, postnatal and adult porcine eyes were grown in suspension sphere culture in serum-free medium containing basic fibroblast growth factor (bFGF) and epidermal growth factor (EGF). Growth curves and BrdU incorporation assays were performed to establish the proliferative capacity of isolated porcine retina-derived RPCs and ciliary epithelium (CE)-derived RSCs. Self-renewal potential was investigated by subsphere formation assays. Changes in gene expression were assayed by reverse transcription polymerase chain reaction (RT-PCR) at different passages in culture. Finally, differentiation was induced by addition of serum to the cultures and expression of markers for retinal cell types was detected by immunohistochemical staining with specific antibodies.

**Results:**

Dissociated cells from embryonic retina and CE at different postnatal ages generated primary nestin- and Pax6-immunoreactive neurosphere colonies in vitro in numbers that decreased with age. Embryonic and postnatal retina-derived RPCs and young CE-derived RSCs displayed self-renewal capacity, generating secondary neurosphere colonies. However, their self-renewal and proliferation capacity gradually decreased and they became more committed to differentiated states with subsequent passages. The expansion capacity of RPCs and RSCs was higher when they were maintained in monolayer culture. Porcine RPCs and RSCs could be induced to differentiate in vitro to express markers of retinal neurons and glia.

**Conclusions:**

Porcine retina and CE contain RPCs and RSCs which are undifferentiated, self-renewing and multipotent and which show characteristics similar to their human counterparts. Therefore, the pig could be a useful source of cells to further investigate the cell biology of RPCs and RSCs and it could be used as a non-primate large animal model for pre-clinical studies on stem cell-based approaches to regenerative medicine in the retina.

## Introduction

Blinding degenerative diseases of the retina, such as retinitis pigmentosa (RP) and age-related macular degeneration (AMD) manifest with different pathologies and have heterogeneous genetic causes. However, they all involve the unifying feature of photoreceptor loss. Similar to the rest of the nervous system, the mammalian retina lacks the capability to self-regenerate in response to damage and there are at present no regenerative therapies available for such retinal diseases [1-4].

Recently, retinal progenitor cells (RPCs) have been identified in embryonic and newborn retina and retinal stem cells (RSCs) in adult ciliary epithelium (CE) of rodents and human [5-12]. These stem cells have the capacity for in vitro self-renewal and differentiation into retinal neurons and glia. Transplanted RPCs and RSCs can survive showing different degrees of integration into the intact and degenerating retina and can differentiate into cells expressing markers characteristic of retinal neurons [11,13-16]. Furthermore, recent studies have indicated that RSCs from adult mammalian CE can be reactivated in vivo upon growth factor administration [17]. A recent report also indicates that retinal neurons derived from transplanted RPCs are functional [18].

Work in rodents has provided an important and necessary step in understanding the cell biology of RPCs and RSCs and in exploring their regenerative potential upon transplantation. However, the rodent eye with its small size, small vitreal space and a low cone/rod ratio is less than ideal to test the efficacy and safety of transplanted stem cells before moving towards human clinical trials. On the other hand, the porcine eye is of similar size and anatomy to the human eye and its retina contains an area centralis rich in cones which resembles the human fovea [19,20]. Moreover, transplantation procedures performed on porcine eyes are comparable to those performed in the clinic [16,21]. Finally, porcine models of retinal degenerative diseases are available [22] and represent an excellent non-primate model system for pre-clinical tests of cell replacement treatments.

Neural precursor and stem cells (NPCs and NSCs) from embryonic and postnatal porcine brain have been isolated, expanded in vitro and tested for xenografts in rat models of Parkinson's disease where they survived and differentiated to form efferent and afferent synapses [23,24]. Conversely, murine RPCs have been xenografted into the porcine retina and shown integration and morphological differentiation [16]. Notably, porcine brain-derived NPCs express specific markers similar to their human counterparts [25]. Whether cells with characteristics of RPCs and RSCs exist in the porcine eye, whether they could be expanded in vitro and whether they could differentiate into specific retinal cell types remains unknown. In this study, we show that the porcine retina and adult CE harbour, respectively RPC and RSC displaying properties of self-renewal and multipotentiality.

## Methods

### Animals

Mixed sex White Landrance pigs were obtained from the Department of Agriculture and Rural Development Northern Ireland, Hillsborough, UK. Embryos (n=30) were obtained from 40-week old gilts (n=3) at 50, 64, and 61 days of gestation. Neonatal pigs (1-3-week old) ranged in weight from approximately 3 kg to 7 kg. Fifteen-week old pigs were 50-55 kg in weight. All animal procedures were performed in compliance with the UK Animals (Scientific Procedures) Act 1986.

### Preparation of retina for histology and immunohistochemistry

Eyes were enucleated from lethally anesthetized pigs at the indicated ages. The cornea and optic nerve were perforated to aid immersion-fixation in 4% (w/v) buffered paraformaldehyde (Sigma-Aldrich, Poole, UK) for 4-18 h at 4 °C. After cryoprotection in 5% (w/v) followed by 30% (w/v) sucrose in phosphate-buffered saline (PBS), ocular tissue specimens were embedded in optimal cutting temperature (OCT) compound (Sakura-Finetek, Zoeterwoude, NL) and snap frozen on dry ice/isopentane. Cryosections (15 μm) were thaw mounted onto Superfrost Plus glass slides (Fisher Scientific, Loughborough, UK) and stored at -80 °C until use. For histology, after haematoxylin and eosin (H &E) staining tissue sections were coversliped with DPX mounting medium and images recorded using a Nikon DXM1200 light microscope (Nikon, Kingston upon Thames, UK).

### Isolation of retinal progenitor cells, retinal stem cells, and neural stem cells from porcine tissues

Eyes were enucleated from pigs at embryonic day (E)60 (n=30), postnatal day (PN)5 (n=13), (PN)21=3 weeks of age (n=9), (PN)150=21 weeks (n=20), 15-weeks (n=14), and 40-weeks (n=3) and placed in artificial cerebral spinal fluid [aCSF: 124 mM NaCl, 5 mM KCl, 1.3 mM MgCl_2_, 26 mM NaHCO_3_, and 10 mM D-glucose, (Sigma-Aldrich, Poole, UK)]. For E60 cultures embryos were obtained from 3 gilts (40 week old). Retinas from 5-7 embryos in a litter were combined to generate a single culture. All other cultures were generated combining tissue from two eyes of an individual animal. The neural retina was first dissected free of the optic nerve. A strip of ocular tissue containing the CE was then dissected after removal of cornea and lens. The CE was transferred into Earle's Balanced Salt Solution (EBSS) containing 2 mg/ml dispase (Sigma-Aldrich, Poole, UK) and incubated for 20 min at 37 °C. After digestion in trypsin mix [aCSF modified to contain 3.2 mM MgCl_2_, 0.1 mM CaCl_2_, 1.33 mg/ml trypsin, 0.67 mg/ml hyaluronidase, and 78 units/ml collagenase (Sigma-Aldrich, Poole, UK)] for 20 min at 37 °C, the CE cells were gently scraped off the basement membrane and the non-epithelial tissue was removed. Cells from the neural retina were isolated after incubation with the same enzymes for 10 min for each digestion. The suspensions containing CE and retina cells were mechanically triturated, and centrifuged at 1000 rpm for 10 min. The supernatants were removed, replaced with serum-free medium (SFM: DMEM/F12 (1:1) with 0.6% glucose, 2 mM glutamine, 5 mM HEPES buffer, 2% B27, 100 units/ml penicillin and 100 units/ml streptomycin) containing 1 mg/ml trypsin inhibitor (all from Invitrogen, Paisley, UK) and the tissue was further mechanically dissociated into single cells using a fire-polished pipette. Cell suspensions were cleared of debris with a 40 μm cell strainer (BD Biosciences, Oxford, UK), centrifuged at 1000 rpm for 10 min and resuspended in SFM. Cells were counted and plated at a density of 2x10^4^ cells/ml in T75 flasks in SFM containing either no exogenous growth factors or epidermal growth factor (EGF), basic fibroblast growth factor (bFGF), and EGF plus bFGF (Invitrogen, Paisley, UK). Cells were allowed to proliferate in suspension to form floating sphere colonies that were counted after 7 days in culture.

Brain NSCs were isolated from E60 pig embryos (n=6). Following removal of the brain, the striatum and periventricular areas were dissected and placed in ice-chilled sterile aCSF. Tissue was digested in PBS containing 1.33 mg/ml trypsin, 78 units/ml collagenase and 0.6% glucose for 10 min at 37 °C. The digest was decanted, replaced with SFM containing 1 mg/ml trypsin inhibitor, and the tissue mechanically dissociated into single cells with a fire-polished pipette. All subsequent preparation steps were the same as described above for RPCs and RSCs. Brain tissue from two pigs were combined to generate a single culture.

### Sphere-forming capacity, expansion, and differentiation

Sphere-forming capacity was employed to assess the rate at which dissociated single cells proliferate to form sphere colonies. Single cells from the CE were plated at a density of no more than one cell per well in 96-well plates (confirmed under microscope after plating) in SFM containing 20 ng/ml EGF and 20 ng/ml bFGF. After 7 days in culture the wells were inspected and scored for the presence or absence of spheres.

Expansion capacity of RPCs and RSCs was assessed by using either suspension sphere or adherent culture conditions. For suspension sphere culture cells were maintained in sphere-forming medium (SFM containing 20 ng/ml EGF and 20 ng/ml bFGF). Every 7 days RPC and RSC spheres were counted, collected, and digested in 0.05% trypsin-EDTA (Invitrogen, Paisley, UK) for 10 min at 37 °C. The cell suspension was then centrifuged at 1000 rpm for 10 min and the enzyme solution replaced with SFM containing trypsin inhibitor. Spheres were mechanically triturated into single cells with a fire-polished pipette and centrifuged at 1000 rpm for 10 min. Single cells were resuspended in sphere-forming medium and plated at a density of 2x10^4^ cells/ml in the same medium.

For adherent culture, dissociated RPC and RSC spheres were cultured in adherent culture medium [SFM containing 20 ng/ml EGF, 20 ng/ml bFGF, 2 μg/ml heparin (Invitrogen, Paisley, UK) and 5% fetal bovine serum (FBS; Invitrogen, Paisley, UK)]. Half volume of culture medium was changed every 2-3 days. After reaching 80-90% confluence, cells were treated with 0.05% trypsin-EDTA for 5 min at 37 °C and neutralized by trypsin inhibitor. Cells were then plated at a density of 5x10^4^ cells/ml in adherent culture medium.

To assay the differentiation capacity of RPCs and RSCs, whole or dissociated spheres (2x10^4^ cells/ml) were plated on poly-D-lysine-coated (Sigma-Aldrich, Poole, UK) glass coverslips in 12-well dishes and incubated in differentiation medium (SFM with 10 ng/ml EGF, 10 ng/ml bFGF, 2 μg/ml heparin and 10% FBS) for 2-3 weeks, with medium changes every 3-4 days.

### Immunostaining

Immunohistochemistry (IHC) was performed on retina cryosections and on cells grown or differentiated on coated glass coverslips. The former were thawed at room temperature and post-fixed in 4% formaldehyde (Sigma-Aldrich, Poole, UK) in PBS for 1 h at room temperature. The latter were fixed in 4% paraformaldehyde (Sigma-Aldrich, Poole, UK) for 20 min at room temperature. After washing, tissue or cells were incubated in antibody blocking buffer [PBS containing 10% (v/v) normal goat serum (NGS), 0.3% Triton X-100, 0.1% NaN_3_ (Sigma-Aldrich, Poole, UK)] for 1 h at room temperature. Slides or coverlips were then incubated in primary antibodies (Table 1) for 48 h at 4 °C. After washing, incubation in fluorescent-conjugated secondary antibody (Alexa Fluor^488^-goat anti-mouse or -goat anti-rabbit, 1:800 in PBS) was performed for 1 h at room temperature followed by washings. Cell nuclei were counterstained with 10 μg/ml propidium iodide (Sigma-Aldrich, Poole, UK) in dH_2_O containing 10 μg/ml RNase A (Invitrogen, Paisley, UK) for 10-20 min at room temperature. After washing, slides were prepared for imaging with antifade mounting medium (Dako, Ely, UK). Negative IHC controls were performed in parallel by omission of the primary antibody. No fluorescent labeling was observed in the negative controls. Immunoreactive cells were visualized and images recorded using an Olympus BX60 fluorescent confocal microscope (Olympus, Europe, Hamburg, Germany).

**Table 1:** List of primary antibodies used and dilutions.

**Primary antibody**	**Host**	**Dilution**	**Source**
Nestin	Mouse	1:400	BD Biosciences
Pax6	Rabbit	1:1,000	Chemicon
β-tubulin III	Mouse	1:100	Chemicon
rhodopsin (4D2)	Mouse	1:100	Kind gift of R. Molday [32]
HuC/HuD	Mouse	1:200	Molecular Probes
recoverin	Rabbit	1:1,000	Kind gift of K. Koch [33]
Islet-1	Mouse	1:500	Developmental Studies; Hybridoma Bank*
calbindin	Rabbit	1:1,000	SWANT
neurofilament-M	Rabbit	1:200	Chemicon
GFAP	Rabbit	1:500	Dako

*The monoclonal antibody developed by T.M. Jessell was obtained from the Developmental Studies Hybridoma Bank developed under the auspices of the NICHD and maintained by The University of Iowa, Department of Biological Sciences, Iowa City, IA-52242.

### 5-Bromo-2-deoxyuridine (BrdU) incorporation and flow cytometry

Cells were cultured for 48 h in the presence of 10 μM BrdU (Sigma-Aldrich, Poole, UK), fixed in 4% paraformaldehyde for 20 min at room temperature and incubated with blocking buffer (PBS containing 10% NGS, 0.3% Triton X-100, and 100 μg/ml RNaseA) for 30 min at 37 °C. Cells were then washed in PBS, incubated with 2N HCl for 20 min at room temperature, and washed with Hanks' Balanced Salt Solutions (HBSS) followed by PBS at room temperature. After overnight incubation at 4 °C with anti-BrdU antibody (1:500, Sigma-Aldrich, Poole, UK) in blocking buffer, cells were washed in PBS, and incubated with fluorescent-conjugated secondary antibody (Alexa Fluor^488^-goat anti-mouse, 1:500) for 1 h at room temperature. Cell nuclei were counterstained with 10 μg/ml propidium iodide for 10 min at room temperature. Immunoreactive cells were visualized and images recorded using an Olympus BX60 fluorescent confocal microscope (Olympus, Europe, Hamburg, Germany).

For quantification of BrdU-positive cells, after incubation with BrdU (see above) cells were trypsinized, washed, blocked and incubated with primary and secondary antibodies as described above and analyzed with a BD FACScalibur flow cytometer (BD Biosciences, Oxford, UK). Samples stained only with secondary antibody were used as negative control.

### Reverse transcription-polymerase chain reaction

Total RNA was extracted from cultured cells at different passages as indicated using the RNeasy Mini kit according to manufacturer instruction (Qiagen, Crawley, UK) followed by in column treatment with DNase I (Qiagen, Crawley, UK). Reverse transcription was performed with Superscript^TM^ II reverse transcriptase and random primers (Invitrogen, Paisley, UK). Amplification of β-actin served as the internal control. The primers and cycling conditions for RT-PCR are shown in Table 2.

**Table 2:** List of primers and cycling conditions for RT-PCR.

**Gene**	**Primer sequence (5'-3')**	**Product size (bp)**
Nestin^1^	F: GGCTTCTCTCAGCATCTTGG	
	R: AAGGCTGGCATAGGTGTGTC	150
β-tubulin III^2^	F: CAGAGCAAGAACAGCAGCTACTT	
	R: GTGAACTCCATCTCGTCCATGCCCTC	250
GFAP^1^	F: TTGACCTGCGACGTGGAGTC	
	R: AGGTGGCGATCTCGATGTCC	225
β-actin^2^	F: CTTCCCCTCCATCGTGGG	
	R: GTGGTACGGCCAGAGGCG	355

^1^Amplification conditions were 1 min/94 °C, 1.5 min/57 °C, 1.5 min/72 °C for 32 cycles. ^2^Amplification conditions were 1 min/94 °C, 1.5 min/56°C, 1.5 min/72 °C for 30 cycles.

## Results

### Localization of nestin-immunoreactive cells in the porcine retina and ciliary epithelium

**Figure 1:**
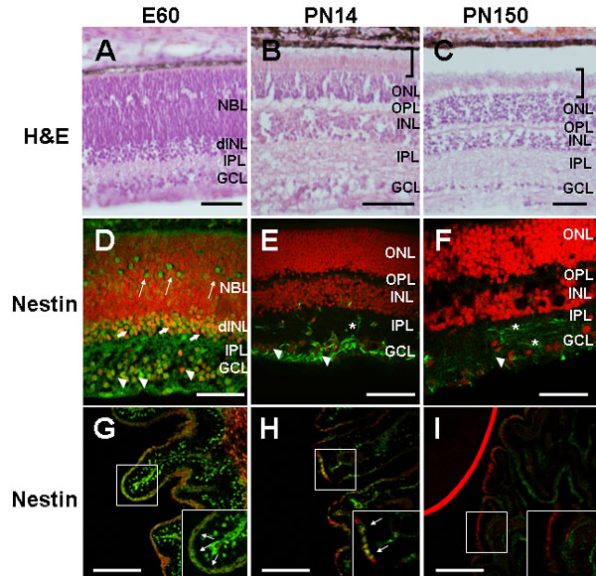
Identification of retinal progenitor cells and retinal stem cells in the developing and mature retina and ciliary epithelium. **A**-**C**: light microphotographs of H&E stained 15 μm retina cryosections. At PN14 (**B**) retinal histology was characteristic of the mature (PN150) retina (**C**) with each of the nuclear and plexiform layers readily identifiable. Mature outer segments of the photoreceptors (brackets) were already evident in the PN14 retina. The apparent detachment of the retina in **C** is a histological artifact. **D**-**F**: confocal fluorescent microphotographs of retina and **G**-**I**: of ciliary body (CB) cryosections (15 μm) immunostained with anti-nestin antibody (green). At E60 nestin immunoreactivity was observed in the ganglion cell layer (GCL; arrowheads), (developing) inner nuclear layer (dINL; thick arrows), neuroblast layer (NBL; thin arrows), and in the inner plexiform layer (IPL). At PN14 (**E**) nestin immunostaining was observed in the GCL (arrowheads) and IPL (asterisk). By PN150 (**F**) nestin immunoreactivity was observed in fibers in the GCL and IPL (asterisks), and in sparse cells in the GCL (arrowhead). At both E60 (**G**) and PN14 (**H**) nestin immunoreactivity was observed in cells distributed within the CB epithelium (thin arrows in insets at bottom). A higher percentage of cells with intense nestin immunostaining were observed in the E60 CB (**G**). Nestin immunoreactivity was not detected in the PN150 CB (**I**). Insets in **G**-**I** represent higher magnification images of the marked areas. Labeled stromal cells in **G** most likely represent migrating precursors of neural crest origin. The red line in I corresponds to propidium iodide (PI) staining in the adjacent lens. Nuclei were counterstained with PI (red). Scale bars: **A**, **G**-**I**, 50 μm; **D**-**F**, 100, **B**-**C**, 200 μm. ONL represents outer nuclear layer, OPL represents outer plexiform layer.

Three ages were selected for collection of porcine retinal tissue: embryonic day (E)60, when the retina is still developing [20,26]; postnatal day (PN)14, when retinal development is mostly complete [26]; and PN150 for mature adult retina. Retinal cryosections were H &E stained or immunolabeled with anti-nestin antibody to visualize the resident pool of immature neuroepithelial cells in the retina and CE (Figure 1). At PN14 (Figure 1B) retinal histology was characteristic of the mature (PN150) retina (Figure 1C) with each of the nuclear and plexiform layers readily identifiable. Mature outer segments of the photoreceptors were already evident in the PN14 retina as compared to adult retina (bracket). At E60 (Figure 1D) nestin immunoreactivity was observed in the majority of cells in the ganglion cell layer (GCL, arrowheads) and developing inner nuclear layer (dINL, thick arrows), in a small population of cells located in the neuroblast layer (NBL, thin arrows), and in fibers in the inner plexiform layer (IPL). At PN14 immunostaining of cells in the GCL (arrowheads) and fibers in the IPL (asterisk) was observed (Figure 1E). By PN150 (Figure 1F) weak nestin immunoreactivity was detected in rare cells in the GCL (arrowhead) and in fibers in the GCL and IPL (asterisks). These observations are in agreement with the presence of nestin immunoreactive cells in the adult human retina previously described [27]. At both E60 (Figure 1G) and PN14 (Figure 1H) nestin immunoreactivity was observed in cell bodies distributed within the ciliary body (CB, thin arrows), the anatomical structure comprising the CE. A higher percentage of cells with intense immunostaining were detected at E60 than in PN14 CB (compare panels G and H). Specific nestin immunoreactivity was not observed in the PN150 CB (Figure 1I). Thus, undifferentiated nestin-immunoreactive cells were present in the developing retina and CB and their proportion decreased as retinal development progressed.

### Isolation and expansion of retina-derived retinal progenitor cells and ciliary epithelium-derived retinal stem cells

**Figure 2:**
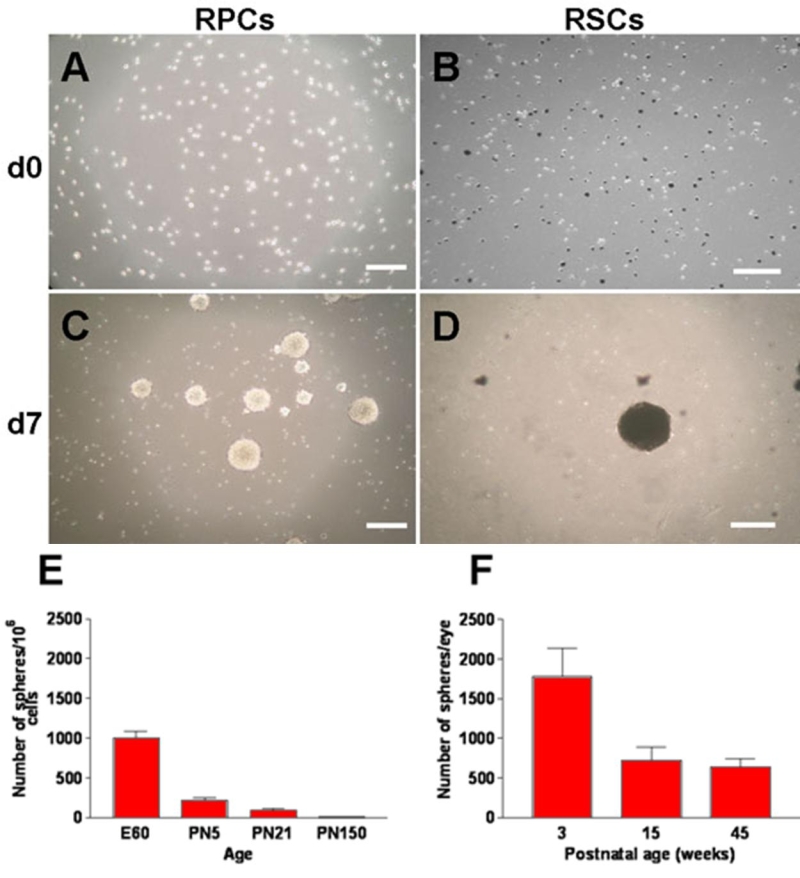
Primary sphere formation in serum-free medium in vitro. **A**: single cells from primary cultures of dissociated E60 retina-derived cells and **B**: of 3 week old ciliary epithelium (CE)-derived cells at day 0. Dissociated CE cultures comprise pigmented and non-pigmented cells (**B**). **C**, **D**: primary sphere colonies at day 7 after plating, showing pigmented spheres in CE-derived RSC cultures (**D**). Scale bars represent 200 μm. **E**, **F**: number of primary spheres formed from retina (**E**) and CE-derived (**F**) primary cultures at different ages. Three week old CE cultures generated more primary spheres than those from 15 and 45 week old pigs (**F**). Data are expressed as mean±SD from three independent experiments.

Ocular tissues from embryonic, newborn, and adult pig eyes were dissected to isolate and culture in vitro RPCs from E60, PN5, PN21, and PN150=21 week old retina and RSCs from 3, 15, and 45 week old CE (Figure 2A,B). Three days after plating, small primary sphere colonies could be observed that continued to expand and, after 7 days, generated relatively large colonies, up to 300 μm in diameter (Figure 2C,D). Spheres derived from dissociation of CE contained both pigmented and non-pigmented progeny (Figure 2D). The number of primary spheres did not increase when the cultures were maintained in culture for another two weeks.

Cells proliferated to form clonal primary spheres in the absence of exogenous growth factors. To establish the effect of mitogens on cell growth, 2x10^4^ 3 week old CE-derived cells were plated in 12 multi-well plates in sphere-forming medium with or without 20 ng/ml EGF and 20 ng/ml bFGF, alone or in combination and cultured for seven days. bFGF increased by 4 times the proportion of cells forming clonal primary spheres in vitro (from 9.7±1.53 spheres/well in SFM, n=3 to 41.3±6.03 spheres/well in SFM+bFGF, n=3). When both EGF and bFGF were added to the culture medium, the number of primary sphere colonies did not increase compared to bFGF alone (42.3±7.37 spheres/well, n=3). The total number of spheres generated from dissociated retinae and maintained in the presence of EGF and bFGF was highest at E60 and decreased with increasing age to less than three spheres generated from 10^6^ dissociated retinal cells at PN150 (Figure 2E). Similarly, the total number of primary spheres generated by cells dissociated from 3 week old CE was greater than those from 15 and 45 week old porcine CE (Figure 2F). The latter two generated a similar number of primary spheres suggesting that the number of cells with potential to proliferate in vitro remains constant throughout adult age. The sphere-forming capacity of 3 week old CE-derived cells was further assayed by plating in sphere-forming medium at a density of one cell per well in individual 96-well dishes. After 7 days in culture, a single sphere was observed in 3 out of 948 wells. This indicated that approximately 0.3% of porcine 3 week old (PN21) CE-derived cells were capable of self-renewal and of forming primary sphere colonies. All subsequent experiments were performed with E60 retina-derived (RPCs) and 3 week old CE-derived cells maintained in cultures enriched with EGF and bFGF.

**Figure 3:**
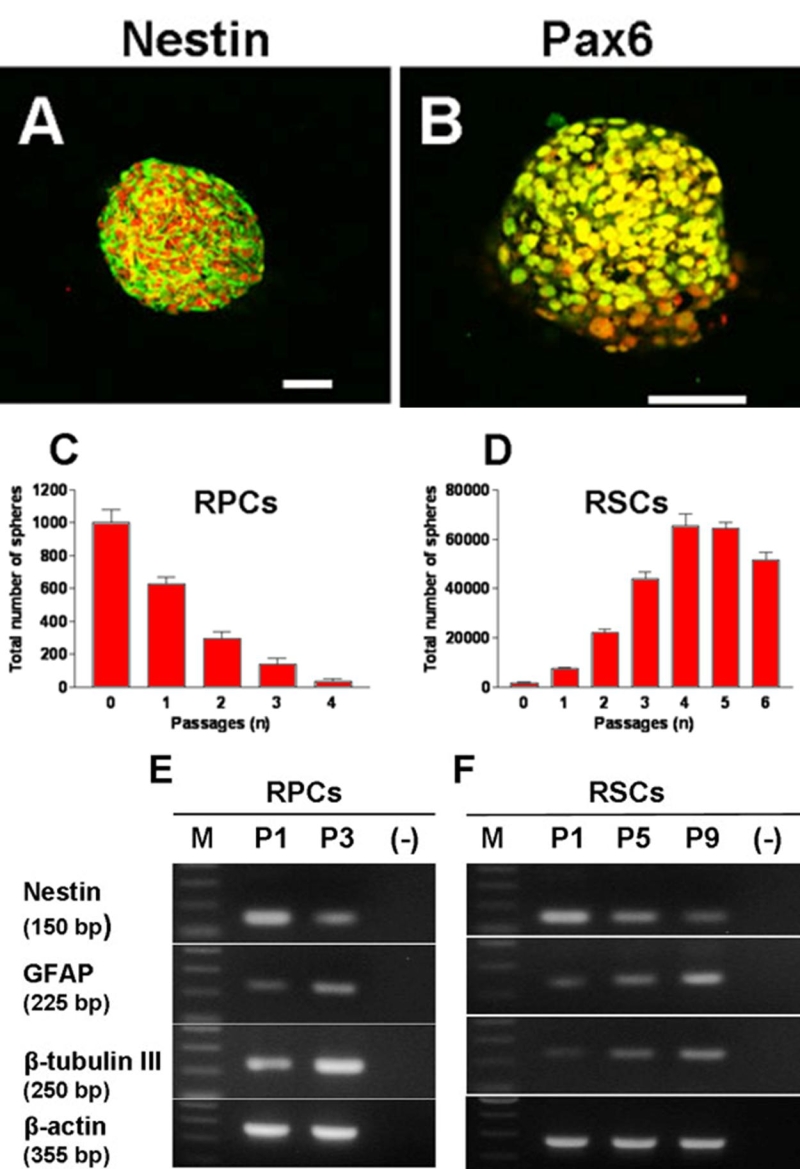
Subsphere formation and gene expression changes with passage. **A**, **B**: subcultured retinal stem cell (RSC) spheres express the undifferentiated retinal cell markers nestin (**A**) and Pax6 (**B**) by immunohistochemistry (IHC). Scale bars represents 100 μm. Nuclei were stained with propidium iodide. **C**, **D**: dissociated retinal progenitor cell (RPC) and RSC spheres generated secondary spheres when grown in suspension. Data are expressed as mean±SD from three independent experiments. **E**, **F**: RT-PCR analysis of RNA from RPC (**E**) and RSC (**F**) spheres at different passages. M indicates molecular weight marker lane. (-) indicates PCR amplification using cDNA synthesis reactions without reverse transcriptase. β-actin was used as internal control.

Immunostaining of RPCs at passage (P)2 and of RSCs at P3 showed that the majority of cells within the RPC and RSC spheres were nestin- and Pax6-positive, revealing their undifferentiated phenotype (Figure 3A,B). To investigate the expandability and self-renewal potential of RPCs and RSCs their sub-sphere-forming capacity was assessed at each passage in culture. Spheres were counted, dissociated into single cells and cultured in sphere-forming medium for seven days before new sphere counts were taken. RSCs from one eye generated on average 1,773±619 primary sphere colonies which could be expanded to 65,267±8,684 sphere colonies within four passages in vitro (Figure 3D). RSC spheres could be maintained in vitro up to P10 (the longest sphere culture attempted). The total number of spheres increased up to P4, and decreased during subsequent passages. The average number of secondary spheres derived from one sphere at P1 was 4.6±1.65, it then dropped gradually to the ratio of one sphere giving rise to less than one sphere at P5 (0.99±0.076) and P6 (0.81±0.128). Furthermore, both the average sphere size and the number of pigmented cells within the spheres appeared reduced with passages (data not shown). Similarly, RPC spheres derived from dissociated E60 retinas could be subcultured in vitro (Figure 3C) up to P4 (the longest sphere culture attempted). However, the total number of spheres gradually decreased with passages and the average number of new spheres formed from dissociation of each single sphere at each passage ranged from 0.6±0.04 at P1 to 0.2±0.19 at P4. Attempts to subculture spheres derived from PN5, PN21, and PN150 retinae were unsuccessful. NSCs isolated from porcine E60 brains were cultured for comparison. As expected, primary spheres derived from brain NSCs generated subspheres continuously at a ratio of one sphere producing approximately eight new spheres at each passage (data not shown).

RT-PCR analysis of mRNA from RPC and RSC spheres at different passages in vitro was performed to investigate changes in expression of undifferentiated and differentiated cell markers. While expression of nestin decreased, expression of β-tubulin III (marker of differentiated neuronal cells) and of glial fibrillary acidic protein (GFAP, marker of differentiated glial cells) increased with increasing passages (Figure 3E,F). Together with the sphere subculture experiment, changes in gene expression suggested that increasing numbers of cells within the spheres had differentiated. This highlighted the limited self-renewal potential of CE-derived RSCs and even more so of retina-derived RPCs.

**Figure 4:**
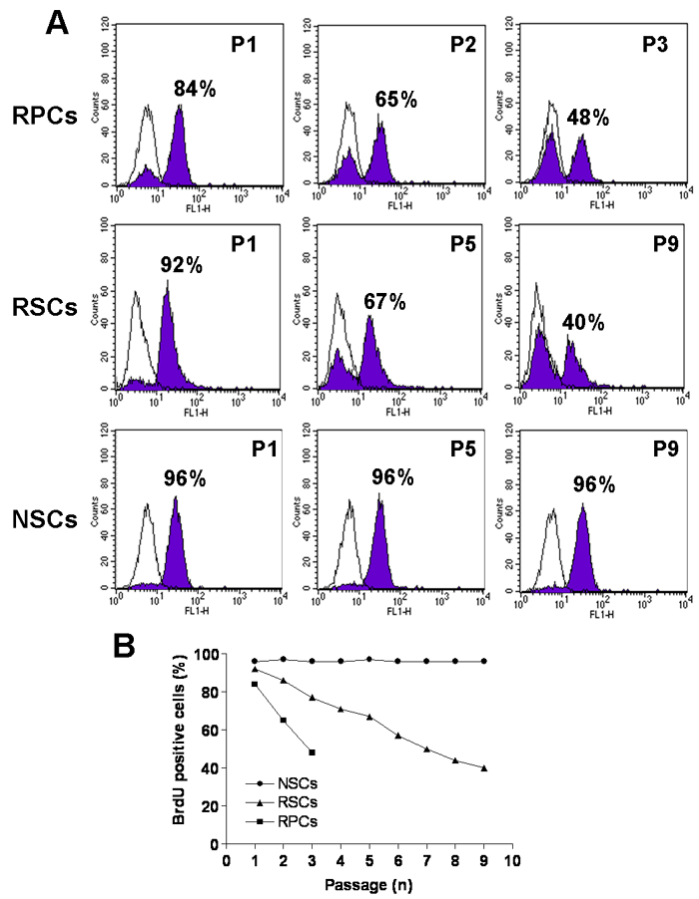
BrdU incorporation of retinal progenitor cell, retinal stem cell, and neural stem cell spheres at different passages. **A**: 84% and 92% of cells within retinal progenitor cell (RPC) and retinal stem cell (RSC) spheres, respectively were positive for BrdU at P1. The proportion of BrdU-positive cells decreased with increasing passages to less than 50% at P3 for RPCs and P9 for RSCs. The proportion of BrdU-positive cells within brain NSC-spheres remained constant at around 96% from P1 to P9. For each plot the shaded profile shows counts of cells after BrdU labeling detected by FACS, the white profile represents counts of control cells reacted with secondary antibody only. Individual values at each passage are plotted in **B**.

To assess if the proliferative capacity of CE-derived RSCs and of retina-derived RPCs changed under suspension sphere culture conditions the percentage of proliferative cells in the spheres was quantified by combining incorporation of the thymidine analog bromodeoxyuridine (BrdU) and FACS analysis (Figure 4). Cultures of porcine embryonic brain NSCs were used as control in this experiment as NSCs from other species are known to proliferate for prolonged periods of time in vitro. Approximately 84% of the cells derived from RPC spheres and 92% of cells derived from RSC spheres at P1 incorporated BrdU (Figure 4A). The percentage of BrdU-positive cells decreased with increasing passages and less than 50% of the cells were labeled with BrdU in cultures of RPCs at P3 and of RSCs at P9 (Figure 4B). On the contrary, the proportion of BrdU-positive cells within brain NSC spheres remained constant at around 96% from P1 to P9.

Monolayer culture conditions have been shown to facilitate expansion of RPCs and RSCs numbers in vitro [11,28]. The expansion capacity of porcine RPCs and RSCs grown in monolayer was compared to that of cells grown as suspended spheres. Approximately 6x10^5^ RPCs were generated within three passages from 10^5^ cells under adherent culture conditions compared to 2.4x10^4^ cells in the suspension sphere cultures. Similarly, more than 5x10^11^ RSCs were generated from 5x10^4^ cells after 10 passages in monolayer culture conditions.

### Differentiation in vitro of retinal stem cells and retinal progenitor cells

**Figure 5:**
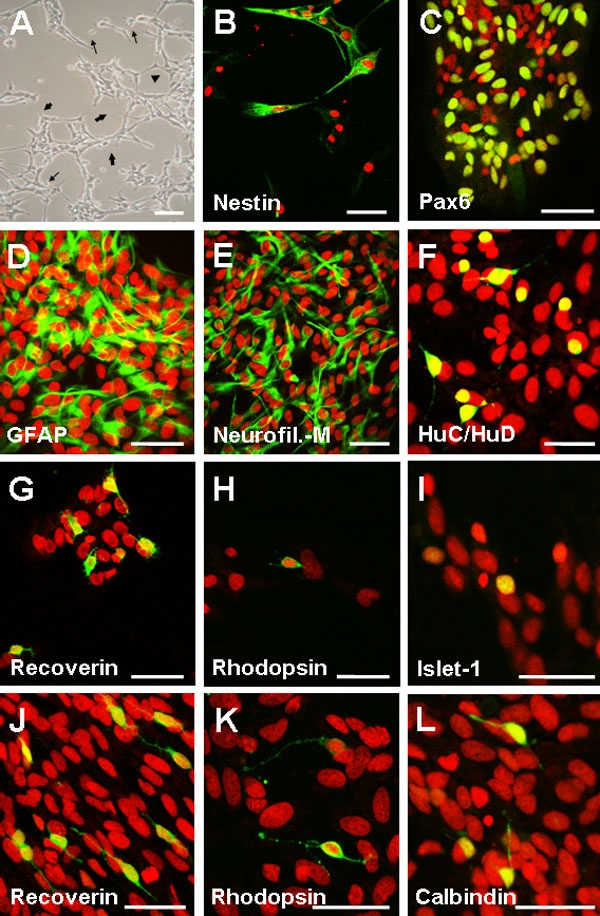
Multipotentiality of retinal stem cells and retinal progenitor cells. Retinal progenitor cells (RPCs) and retinal stem cells (RSCs) were plated on coverslips coated with poly-D-lysine and incubated in differentiation medium for 2 weeks. **A**: phase contrast microphotograph of serum-treated RPCs showing cells with small bodies and elongated dendritic processes (thin arrows), some apparently connected (thick arrows) as well as cells with large, polygonal shapes (arrowhead) to indicate morphological changes associated with neuronal and glial differentiation. **B**-**I**, **L**: RSCs maintained in differentiation medium for 2 weeks were fixed and immunostained with antibodies to: nestin (**B**); Pax6 (retinal progenitors, amacrine cells; **C**); GFAP (glial cells; **D**); neurofilament-M (RGCs, interneurons; **E**); HuC/HuD (horizontal, amacrine cells; **F**); recoverin (cone and rod photoreceptors; **G**); rhodopsin (rod photoreceptors; **H**); Islet-1 (bipolar, amacrine cells **I**); and calbindin (horizontal, amacrine, RGCs; **L**). **J**-**K**: RPCs maintained in differentiated medium for two weeks were fixed and immunostained with antibodies to: recoverin (**J**), and rhodopsin (**K**). Cells expressing the same marker differentiated in clusters. Thus, microphotographs of recoverin and rhodopsin immunostaining in RPCs and RSCs are not for quantitative comparison and are not representative of the counts reported in Figure 6. Nuclei were stained with propidium iodide. Scale bars: **A**, **C**, and **F** represents 100 μm; **B**, **D**-**E**, and **G**-**K** represents 50 μm.

**Figure 6:**
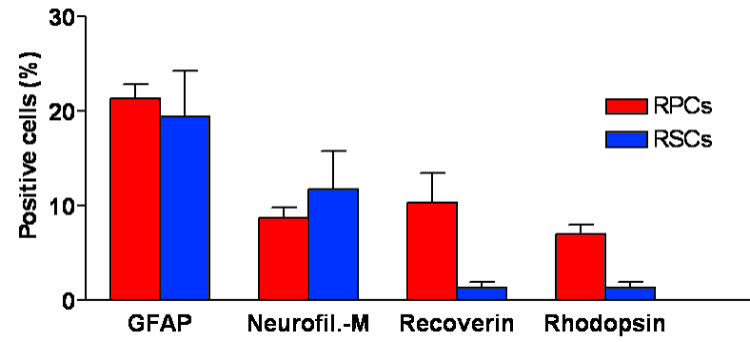
Quantification of E60 retina-derived retinal progenitor cells and 3 week old ciliary epithelium-derived retinal stem cells displaying distinct immunoreactivity after serum-induced differentiation in vitro. Dissociated retinal progenitor cell (RPC) and retinal stem cell (RSC) spheres were incubated in differentiation medium for two weeks, fixed, and immunostained with the indicated antibodies. Quantification was performed by recording the number of immunopositive cells over the number of nuclei counterstained with PI in random fields. Two hundred-1,000 cells for each immunostaining reaction for each culture were counted. Differentiated cells manifested retinal neural phenotypes in different proportions as indicated. A relatively large percentage of cells in both cultures displayed GFAP immunoreactivity. Data represent the mean±SD of three independent experiments.

Under differentiation conditions RPCs at P2 and RSCs at P3 showed morphological hallmarks of neural differentiation and expressed retinal neuronal and glial markers (Figure 5). Some cells had a small soma and several long, thin cell processes (Figure 5A, thin arrows), often connected to each other (thick arrows). A small number of cells were large and polygonal in shape (arrowhead). Expression of undifferentiated and retinal cell-specific markers was investigated by IHC with specific antibodies (Figure 5B-L). Approximately 65-70% of RSCs initiated differentiation as indicated by the number of nestin-negative cells counted (Figure 5B). The coincidental expression of Pax6 in retinal progenitors and differentiated amacrine cells makes it difficult to establish the degree of differentiation using this marker which resulted positive in 32±4.7% of cells (Figure 5C). Differentiated RSCs were immunoreactive for the glial cell marker GFAP (19±4.9%, Figure 5D) and for the neuronal marker neurofilament-M (12±4.0%, Figure 5E). Rare cells (less than 1%) expressed the cone and rod photoreceptor marker recoverin (Figure 5G), the rod photoreceptor-specific marker rhodopsin (Figure 5H), the marker of newborn horizontal, amacrine, and RGCs HuC/HuD (Figure 5F), the interneuron and RGC marker Islet-1 (Figure 5I), and the horizontal cell marker calbindin (Figure 5L). Cells immunoreactive for a specific antibody tended to appear in clusters. Thus, microphotographs document the quality of the immunostaining rather than the proportion of labeled cells. For comparison, E60 retina-derived RPCs at P2 were also differentiated in the presence of serum (see representative immunostaining with the antibodies against recoverin and rhodopsin in Figure 5J and K, respectively) and the proportion of cells immunopositive with retina-specific markers was counted (Figure 6). CE-derived RSCs seemed to possess the potential to differentiate along retinal neuronal and glial cell lineages in vitro in percentages that were comparable to those obtained for E60 retina-derived RPCs (compare GFAP, and neurofilament-M). However, the proportion of cells expressing the photoreceptor markers recoverin and rhodopsin was lower in CE-derived differentiated RSC cultures.

## Discussion

The present study describes the isolation and initial in vivo and in vitro characterization of porcine retina-derived RPCs and CE-derived RSCs. The porcine eye was chosen because of its similar size, anatomy, and histology to the human eye [19,20]. Furthermore, retinal development in the pig shows substantial similarities to human retinal development [26]. These characteristics make the pig an ideal pre-clinical animal model for transplantation experiments.

Our data indicates that the highest yields of RPCs and RSCs can be obtained from dissociated E60 retina and 3 week-old CE, respectively. Porcine RPCs and RSCs displayed properties similar to those isolated from rodent and human. RPCs were derived from adult (PN150) retina at a frequency of approximately 1:3.5x10^6^, which is comparable to the 1:2.0-3.0x10^6^ ratio obtained for adult human RPCs [12]. Similarly, RSCs were derived from porcine 3 week-old CE at a frequency of approximately 1:350, comparable to the 1:600 ratio in human [11]. Together these data indicate that the proportion of RPCs in the retina and of RSCs in the CE are conserved between pig and human. In apparent contrast with the data on human CE-derived RSCs [11], our data showed a decrease in the frequency of RSCs obtained from adult (15 and 45 week old) CE compared to newborn. This difference reflected the presence of higher proportions of nestin-positive cells in the porcine CE at two weeks (PN14) compared to 21 weeks (PN150). The pool of undifferentiated cells at PN14 might be a transitional, short lived pool which persists only shortly after birth and it might be a peculiarity of the developmental process in the pig which requires further investigation.

Under sphere-forming culture conditions, the expansion capacity of CE-derived RSCs was initially comparable to that of brain-derived NSCs but decreased with increasing passages. On the other hand, retina-derived RPCs exhibited a more restricted expansion capacity when compared to CE-derived RSCs and could not be maintained for more than a few passages in suspension sphere cultures. This study employed for the first time BrdU incorporation assay in association with flow cytometry to quantify the number of proliferating RPCs, RSCs and NSCs across different passages. Besides establishing a quantitative approach to determine proliferation in culture, these experiments showed that the proportion of cells within the RPC and RSC spheres that synthesized DNA during a 48 h period decreased with time in culture while the proportion of proliferating brain-derived NSCs remained constant. This suggests that some of the cells within the RPC/RSC spheres had stopped proliferating and had undergone differentiation, or had longer doubling times at late passages in culture. Gene expression analysis suggested that the decrease in cell proliferation was in fact associated with an increase in cell differentiation. These results support the notion that RPCs and RSCs exhibit a restricted self-renewal potential compared to NSCs [8,29]. Whether the limited self-renewal is an intrinsic characteristic or it is determined by culture conditions remains to be established. Notably, the expansion capacity of porcine RPCs and RSCs maintained in monolayer cultures was increased, as it has been shown also for rodent and human cells [7,11,28]. Conclusive evidence awaits the identification of markers that unequivocally distinguish between progenitor and stem cell populations.

Assessing the differentiated phenotype of porcine retina-derived RPCs and CE-derived RSCs had two scopes: to establish their multipotentiality and to investigate their potential to generate retinal cell types which would render them useful for transplantation experiments. Since porcine CE-derived RSCs have the potential to give rise to different retinal cell phenotypes, but predominantly earlier-born (neurofilament-M-immunoreactive neurons), it implies that CE-derived RSCs possess properties of early RPCs. This hypothesis is also supported by a recent report indicating that mouse CE-derived RSCs have more characteristics (e.g. proliferative capacity, potential to generate retinal cell phenotypes, and gene expression pattern) in common with early retinal progenitors than late retinal progenitors [30]. This report also suggests that CE-derived RSCs may be a residual population of stem cells from the optic neuroepithelium, representing a stage antecedent to retinal progenitors. This could be the case also for porcine CE-derived cells. However, whether the CE-derived RSC population is composed of pure early progenitor cells or of heterogeneous subsets of progenitors with distinct competencies still remains to be established since CE-derived RSCs also gave rise to cells expressing markers for late born neurons (e.g. GFAP for glial cells, rhodopsin for rod photoreceptors, and recoverin for cone and rod photoreceptors). Ultimately, the low proportion of rhodopsin-positive cells observed under the present differentiation protocol is a limitation that needs to be addressed in view of potential therapeutic approaches. Recently, the adoption of "priming" protocols has generated high yields of mouse retina-derived RSCs committed to the photoreceptor fate [31]. Similar approaches could be applied to drive photoreceptor-specific commitment from porcine CE-derived RSCs.

In summary, RPCs and RSCs from the porcine eye can be expanded in culture, differentiate in vitro to express markers specific to retinal cell types and show remarkable similarity to their human counterpart. The availability of a pre-clinical model (the pig) whose eye is comparable in size, anatomy, and histology to the human eye can benefit the advancement of the efforts leading to RPC and RSC transplantation in the diseased human eye. Our study opens the way to further characterization of porcine-derived RPCs and RSCs in vitro to investigate the molecular mechanisms regulating their proliferation and differentiation. In the future, in vivo allotransplantation of porcine RSCs using available porcine models of retinal degeneration [22] will reveal their capacity to survive, integrate and differentiate to promote cell repair. Furthermore, the use of the pig model would facilitate pre-clinical development of surgical procedures which could be directly utilized for cell transplantations in the diseased human retina.
